# Supplementary material for: ABCA3 Deficiency—Variant-Specific Response to Hydroxychloroquine
Source: Int J Mol Sci. 2023 May 3;24(9):8179. doi: 10.3390/ijms24098179 (PMC10179277; doi:10.3390/ijms24098179)
Supplement: Supplementary file 1 [file ijms-24-08179-s001.zip › ijms-2368342-supplementary.pdf]

SUPPLEMENT

ABCA3 Deficiency—Variant-Specific Response to Hydroxychloroquine

Xiaohua Yang [1], Maria Forstner [1], Christina K. Rapp [1], Ina Rothenaigner [2], Yang Li [1,3], Kamyar Hadian [2] and Matthias Griese [1,*]

[1] Dr. von Haunersches Kinderspital, University of Munich, German Center for Lung Research, Lindwurmstr. 4a, 80337 Munich, Germany; xiaohua.yang@med.uni-muenchen.de (X.Y.); maria_elisabeth.forstner@med.uni-muenchen.de (M.F.); christina.rapp@med.uni-muenchen.de (C.K.R.); liyanggzyx@outlook.com (Y.L.)

[2] Research Unit Signaling and Translation, Helmholtz 'Zentrum München, Ingolstädter Landstr. 1, 85764 Neuherberg, Germany; ina.rothenaigner@helmholtz-munich.de (I.R.); kamyar.hadian@helmholtz-munich.de (K.H.)

[3] Medical College, Chongqing University, 400044 Chongqing, China

* Correspondence: matthias.griese@med.uni-muenchen.de; Tel.: +49-89-4400-57870; Fax: +49-89-4400-57872

Table S1. Clinical data collected in this study

General information	Gender (male/female)
	Gestational age (weeks)
	Birthweight (grams)
	Age at deaths (years)
	Age at disease onset (years)
	Age when HCQ therapy was started (years)
	Age at initial assessment of HCQ therapy (years)
	Age at the latest assessment of HCQ therapy (years)
Gene information	*ABCA3* variants
Respiratory information	Respiratory symptoms (tachypnoea, cough) (yes/no)
	Respiratory rate (per minute)
	SpO_2 (%)
	Lung function (FVC % predicted)
Clinical management	Oxygenation strategy (mechanical ventilation, high-frequency oscillatory ventilation, continuous positive airway pressure, oxygen flow, or free of O_2 supply)
	Lung transplantation (yes/no, age)
	Medication (HCQ, steroids, azithromycin, cyclosporine A)

Table S2. Definition of a change in respiratory status of the patients at the initial or last assessment of the HCQ response (*in vivo*)

	Improved (scored as 1)	Same (scored as 0)	Deteriorated (scored as -1)
Respiratory symptoms (tachypnoea, cough)	Remission of tachypnoea: respiratory rate (< 40/min in children older than 3 years, < 50/min in children 1 to 3 years, and < 60/min in newborns); or Remission of cough	Same respiratory rate (change < 10/min); or Same frequency of cough (based on clinical record)	New occurrence of tachypnoea: respiratory rate (≥ 40/min in children older than 3 years, ≥ 50/min in children 1 to 3 years, ≥ 60/min in newborns); or new occurrence of cough; or Respiratory rate increased (> 10/min); or Patient died; or Lung transplantated
Oxygenation strategy	From invasive ventilation to non-invasive ventilation; or Reduction of oxygen flow (Δ more than 0.5 L/min); or Increased SpO_2 (> 5%) with the same oxygen strategy; or From oxygen-dependent to free of O_2 supply	Same oxygenation strategy	From non-invasive ventilation to invasive ventilation; or Higher oxygen flow (Δ more than 0.5 L/min); or Lower SpO_2 (> 5%) with the same oxygen strategy; or From oxygen-free to oxygen-dependent

Table S3. Characteristics of patients included into the study

General characteristics	Complete cohort (n = 39)	Improved or unchanged (n = 19)	Initially improved, then deteriorated (n = 5)	Deteriorated (n = 15)
Female (%)	18 (46.1)	8 (42.1)	1 (20.0)	9 (60.0)
SGA (%)	6 (15.4)	1 (52.6)	0	5 (33.3)
Preterm infants (%)	3 (7.7)	1 (5.2)	0	2 (13.3)
Caucasian (%)	33 (84.6)	19 (100.0)	5 (100.0)	9 (60.0)
Homozygous mutation (%)	10 (25.6)	3 (15.8)	2 (40.0)	5 (33.3)
Null/null mutation (%)	3 (7.7)	0	0	3 (20.0)
Null/hypomorphic variants mutation (%)	10 (25.6)	5 (26.3)	0	5 (33.3)
Hypomorphic/hypomorphic mutation (%)	28 (71.8)	16 (84.2)	5 (100.0)	7 (46.7)
Age at death (years)	0.4 (0.1- 5.5)	None	0.9 (0.4-5.5)	0.2 (0.1-0.6)
Age at disease onset (years)	0 (0-5.1)	0 (0-5.1)	0	0 (0-3.5)
Age when HCQ therapy was started (years)	0.3 (0.1-15.8)	2.0 (0.1-15.8)	0.2 (0.1-2.9)	0.1 (0.1-12.8)
Age at initial assessment of HCQ therapy (years)	0.8 (0.1-17.1)	4.0 (0.2-17.1)**	0.2 (0.2-3.0)	0.4 (0.1-13.5)
Age at the latest assessment of HCQ therapy (years)	1.7 (0.3-13.0)	6.0 (0.4-13.0)*	0.6 (0.4-5.5)	0.3 (0.3-0.8)

SGA: Birth weight below the 10th percentile for the gestational age and sex according to Fenton Growth Chart 2013.
Data are shown as median (minimum-maximum).
Improved *vs.* Deteriorated: * p value 0.0332, ** p value 0.0021.

Table S4. Response to HCQ *in vitro**

Variants	% WT-like cells	Proteolytic cleavage of ABCA3	Volume of $ABCA3^+$ vesicle	Transport of propargyl-choline into $ABCA3^+$ vesicle	Average score of response to HCQ *in vitro*
M760R	0	0	0	0	0
Q215K	0	0	0	0	0
G571R	0	ND	ND	ND	0
P32S	0	ND	ND	ND	0
P248S	0	ND	ND	ND	0
C611R	0	ND	ND	ND	0
G1421R	0	2	0	0	0.5
V1399M	0	2	0	0	0.5
G1314E	0	ND	ND	ND	0
A1046E	0	2	2	2	1.5
G202R	1	ND	ND	ND	1
F1077I	1	ND	ND	ND	1
E1364K	1	0	2	2	1.25
D953H	1	2	2	2	1.75
Q1045R	2	2	2	2	2
E292V	2	2	2	2	2

*Listed were the results from the different assays performed (Fig. 3 and Fig 4). The data were coded as 0, no response (no change from defective (> -3 nSD) range, or no change from impaired (> −1nSD to < −3 nSD) range); and as 1, partial response (change from defective range into impaired range); and as 2, complete response (change into normal (< −1 nSD) range. ND: not done in this study.

Table S5. Overview of the variants' response to HCQ *in vitro* and clinical outcomes *in vivo*

		Variant			Score based on ACMG guideline		Respiratory outcomes of patients (rating in each assessment -1, 0, 1, X (no last assessment[§])				
Reference	Pat ID	Allele 1 (score)	Allele 2 (score)	Sum score of response *in vitro* of two *ABAC3* variants	Allele 1 (score)	Allele 2 (score)	Initial assessment	Last assessment	Average score	Response to HCQ *in vivo* (Fig. 2)	Concomitant medication
KLR	1	p.D953H (1.75)	p.F1077I (1)	2.75	5	5	1	1	1	+	Steroids
KLR	2	p.P248S (0)	p.P248S (0)	0	5	5	1	0	0.5	+	Steroids, Azithromycin
[1]	3	p.H778R (ND)	p.L1252P; p.A1528V; c.447+11C>T (ND)	ND	3	4	1	1	1	+	Steroids
KLR	4	p.R208W (ND)	c.3863-98C>T (ND)	ND	4	4	1	1	1	+	Steroids, Azithromycin, Cyclosporine A
KLR	5#	p.E765* (ND)	p.E292V (2)	ND	5	3	1	1	1	+	None
KLR	6	p.W308R (ND)	p.W308R (ND)	ND	4	4	Lung transplantation	-	−1	−	None
KLR	7	p.Q1045R (2)	p.Q1045R (2)	4	5	5	1	X	1	+	Steroids, Azithromycin, Cyclosporine A

KLR	8	p.V1399M (0.5)	p.V1399M (0.5)	1	5	5	1	Died	0	+/−	Steroids, Azithromycin
KLR	9	p. E292V (2)	p.L1105F (ND)	ND	3	4	1	1	1	+	None
KLR	10	p. G964S (ND)	p.R1482W (ND)	ND	4	4	1	X	1	+	None
KLR	11	p.K537R (ND)	p.K537R (ND)	ND		4	1	X	1	+	Steroids, Azithromycin
KLR	12	p.S1262G (ND)	-	ND	3	4	1	1	1	+	Steroids, Azithromycin
KLR	13	p.E292V (2)	p.E1364K (1.25)	3.25	3	5	1	1	1	+	Azithromycin
[2]	14	p.T1114A (ND)	p.W1148* (ND)	ND	5	5	1	1	1	+	Steroids
[3]	15#	p.D507Efs *2 (ND)	p.D696N (ND)	ND	5	4	1	0	0.5	+	Steroids, Azithromycin
[4]	16	p.K914M (ND)	p.L1238_ E1239ins G[6] (ND)	ND	4	5	1	1	1	+	None
KLR	17	p.G1314E (0)	p.P32S (0)	0	5	5	1	Died	0	+/-	Steroids
KLR	18#	p.P969S (ND)	p.D1439G fs*11 (ND)	ND	4	5	-1	−	−1	−	Steroids
KLR	19#	p.A1046E (1.5)	c.4360-1G>C (ND)	ND	5	5	Died	−	−1	−	Steroids
KLR	20	p.G1314R (ND)	p.G1314R (ND)	ND	5	5	1	Died	0	+/−	None

KLR	21##	p.S536Pfs *10 (ND)	p.V1303Sf s*43 (ND)	ND	5	5	Died	−	−1	−	Steroids, Azithromycin
KLR	22	p.G1421R (0.5)	p.Q1045R (2)	2.5	5	5	Died	−	−1	−	Steroids, Azithromycin
KLR	23#	p.Asn104 Thrfr*47 (ND)	p.P246L (ND)	ND	5	5	Died	−	−1	−	None
KLR	24#	p.Q233* (ND)	p.R280C (ND)	ND	5	5	Died	−	−1	−	Steroids
KLR	25##	p.R1333G fs*24 (ND)	p.R1333G fs*24 (ND)	ND	5	5	Died	−	−1	−	Steroids, Azithromycin
KLR	26	p.C611R (0)	p.G202R (1)	1	5	5	1	Died	0	+/−	Steroids, Azithromycin
[5]	27#	p.V1615G fs*15 (ND)	p.R194G (ND)	ND	5	5	Died	−	−1	−	Steroids
[5]	28#	p.V1615G fs*15 (ND)	p.R194G (ND)	ND	5	5	Died	−	−1	−	Steroids
[6]	29	p.R1612P (ND)	p.L798P (ND)	ND	4	4	Died	−	−1	−	Steroids
[7]	30	p.D1149N (ND)	p.D1149N (ND)	ND	4	4	Died	−	−1	−	Steroids, Azithromycin
[8]	31#	p.R1561* (ND)	p.E690G (ND)	ND	5	5	1	1	1	+	Steroids
KLR	32	p.E292V (2)	p.G571R (0)	2	3	5	1	1	1	+	None
KLR	33##	p.R1561* (ND)	p.R1561* (ND)	ND	5	5	Lung transplantation	−	−1	−	None

KLR	34	p.P246L (ND)	p.L1104R (ND)	ND	4	4	-1	−	−1	−	None
KLR	35	p.V1399M (0.5)	p.V1399M (0.5)	1	5	5	Died	−	−1	−	None
KLR	36#	p.E292V (2)	p.R998Pfs *11 (ND)	ND	3		1	X	1	+	None
KLR	37	p.E292V (2)	p.P1301L (ND)	ND	3	4	1	−1	0	+/−	None
KLR	38	p.E292V (2)	p.P248L (ND)	ND	3	5	1	0	0.5	+	None
KLR	39	p.E292V (2)	p.P245L; p.P1301L (ND)	ND	3	4	1	X	1	+	None

ND: not done in this study. # null/hypomorphic variants. ## null/null variants.
[§]Reasons for no last assessment: those 5 patients had no new follow-up yet during our research period.
Sum score of response *in vitro* of two *ABCA3* variants ≥ 2: responsive to HCQ treatment *in vitro*.
Average score of respiratory outcomes of patients > 0: responsive to HCQ treatment *in vivo*.

LEGENDS TO THE SUPPLEMENT FIGURES

Figure S1

Overview of missense *ABCA3* variants selected for experiments *in vitro.*

Green indicated missense variants in this cohort. Red indicated missense variants included in high-content screening (Figure. 3). Yellow indicated missense variants included in western blot and immunofluorescent staining (Figure. 4). Purple indicated missense variants included in co-staining with ABCA3-HA and CD63 / Calnexin (Figure 5, Figure. S6).

Figure S2

WT cells were treated with phenol red free RPMI-1640 + 10% FBS (nt), H_2O, 5 μM, 10 μM, 20 μM, 40 μM of HCQ for 4h, 24h, 48h and 72h. Cell viability was assessed by quantification of the specific cleavage of yellow XTT tetrazolium salt to orange formazan in the presence of phenazine methosulfate. Absorbance at 450 nm was measured using a spectrophotometer (n=3). Results (% WT nt) were shown as means + S.E.M. No significant difference was detected among different treatments at each specific time. *Concentration of HCQ > 40 μM was toxic to A549 cells according to Li et al (2018) and preliminary experiments in our groups.

Figure S3

A549 cells stably expressing WT or mutated ABCA3-HA were treated with RPMI-1640+10% FBS (-) or RPMI-1640 + 10% FBS added HCQ 10 μM (+) for 24 hours. Proteolytic cleavage of ABCA3-HA was analyzed by western blot. Densitometric quantification of protein amount in each band (190 kDa and 170 kDa) was performed using Image J.

Figure S4

A549 cells stably expressing WT or mutated ABCA3-HA were incubated with propargyl-choline (100 μM) and then treated with RPMI-1640+10% FBS (nt) or RPMI-1640 + 10% FBS added HCQ 10 μM (HCQ) for 24 hours. Confocal microscopy images of cells stained for ABCA3-HA were shown. Scale bar represents 10 μm. Confocal microscopy images of cells stained for ABCA3-HA were shown. Scale bar represents 10 μm. ABCA3-HA was in green. Propargyl-choline was in red. DAPI was in blue.

Figure S5

Response of 16 ABCA3 variants to HCQ *in vitro* assessed by high-content screening method (statistical analysis). A549 cells stably expressing WT or mutated ABCA3-HA were treated with RPMI-1640+10% FBS (nt) or RPMI-1640 + 10% FBS added HCQ 10 μM, 30 μM, 40 μM for 24 hours. After fixation and staining with fluorescent DsRed 555 (anti-HA), percentage of ABCA3-HA transfected wild type like cells (% WT-like cells) were analyzed with high-content screening assay. Results (% WT nt) were shown as means + S.E.M. * indicates p value 0.0332, ** indicates p value 0.0021, *** indicates p value 0.0002, **** indicates p value < 0.0001.

Figure S6

A549 cells stably expressing WT or mutated ABCA3-HA were treated with RPMI-1640 + 10% FBS (no treatment) or RPMI-1640 + 10% FBS added HCQ 10 μM (HCQ 10 μM) for 24 hours, and then stained for ABCA3-HA and ER marker calnexin. ABCA3-HA was in green. Calnexin was in red. DAPI was in blue. Scale bar represents 20 μm.

Figure S1

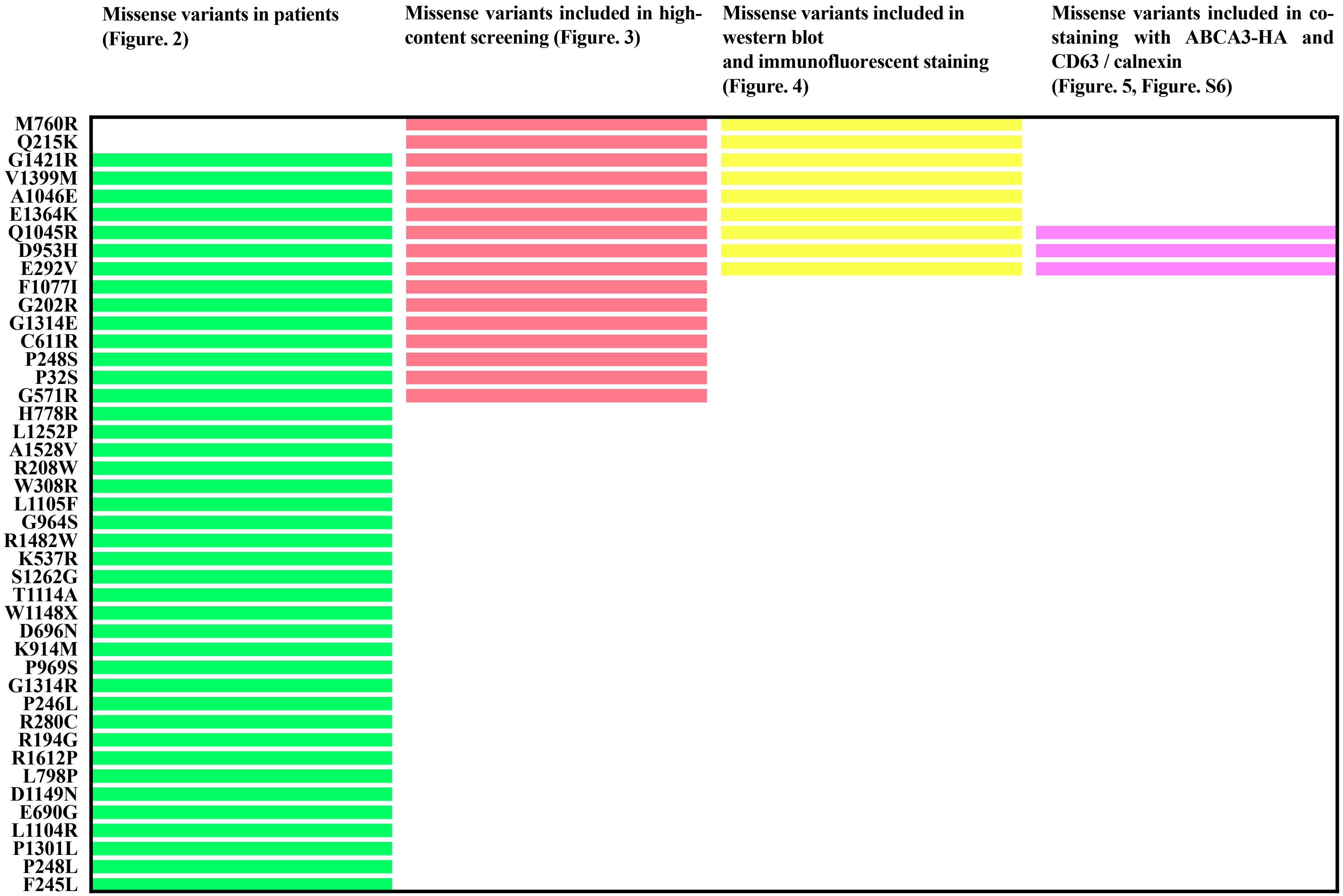

Overview of *ABCA3* missense variants selected for experiments *in vitro*

Figure S2

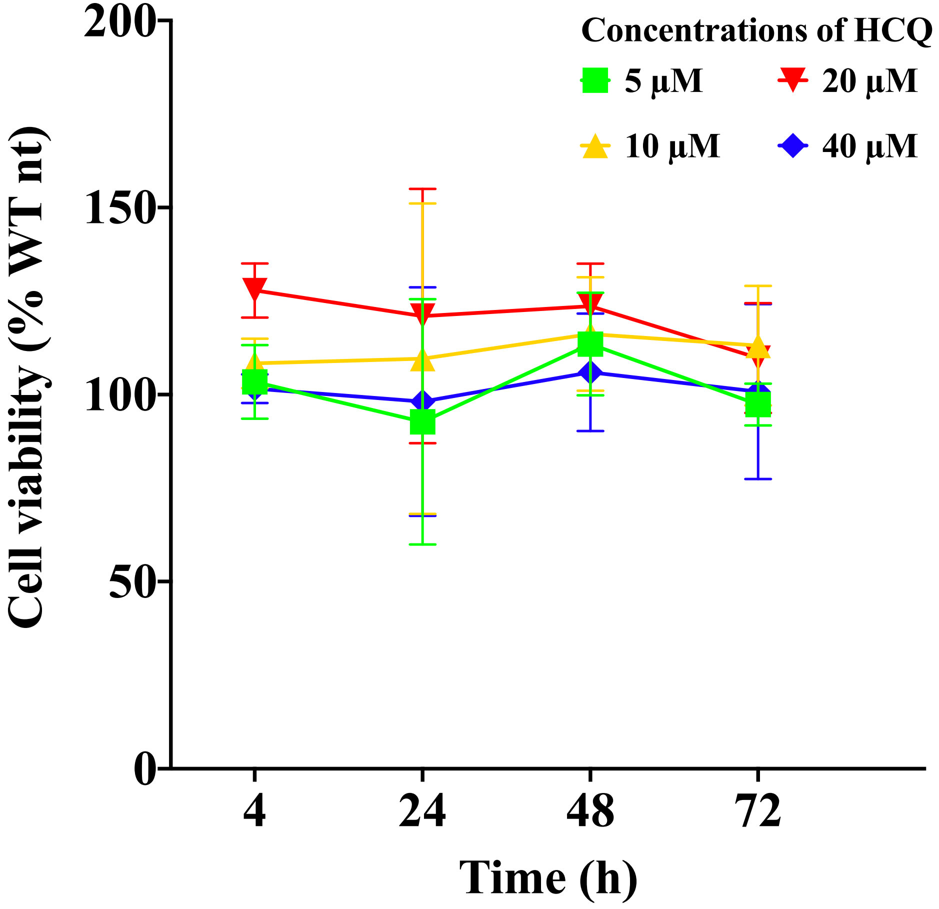

Concentrations of HCQ
5 µM
20 µM
10 µM
40 µM
Cell viability (% WT nt)
200
150
100
50
0
4
24
48
72
Time (h)

Figure S3

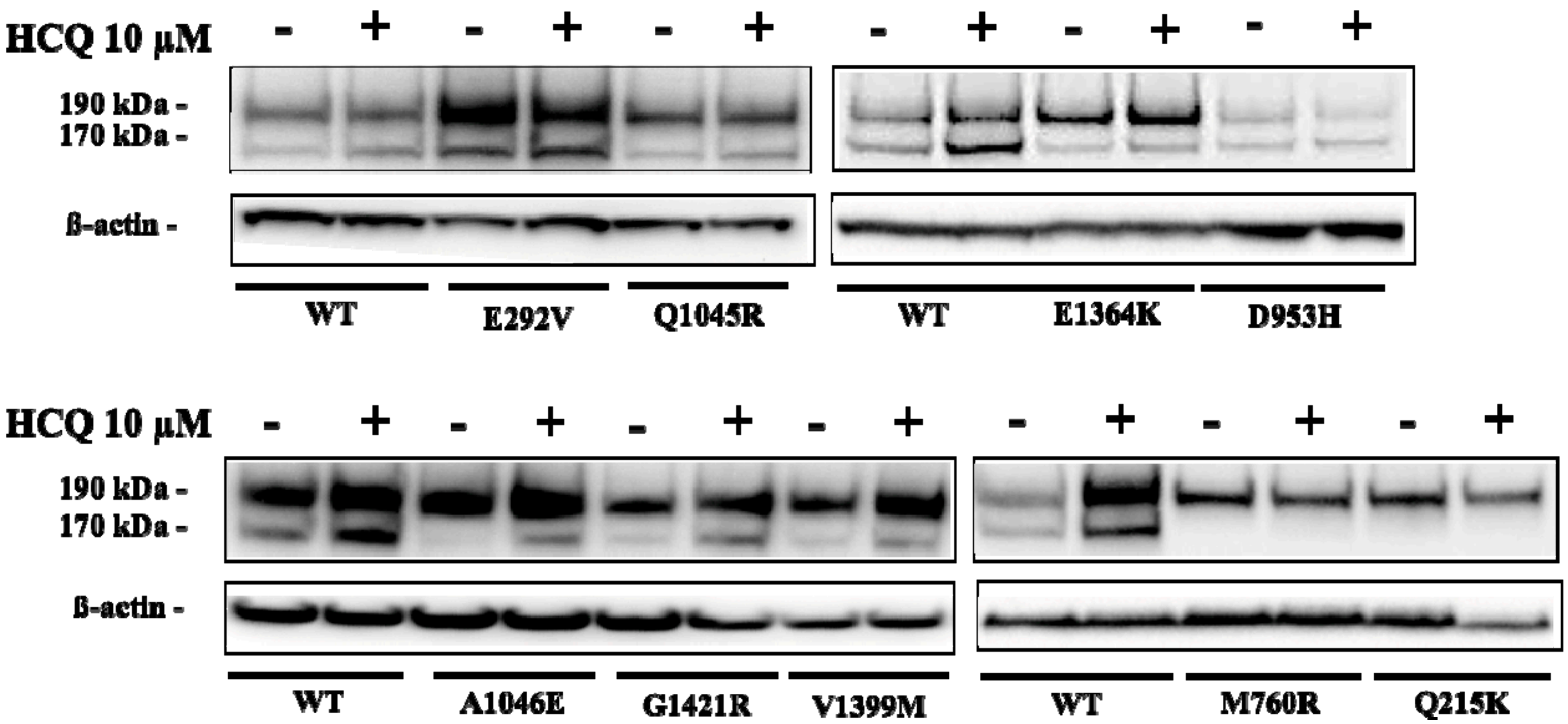

HCQ 10 µM - + - + - + - + - + - +
190 kDa -
170 kDa -
ß-actin -
WT
E292V
Q1045R
WT
E1364K
D953H
HCQ 10 µM - + - + - + - + - + - + - +
190 kDa -
170 kDa -
ß-actin -
WT
A1046E
G1421R
V1399M
WT
M760R
Q215K

Figure S4

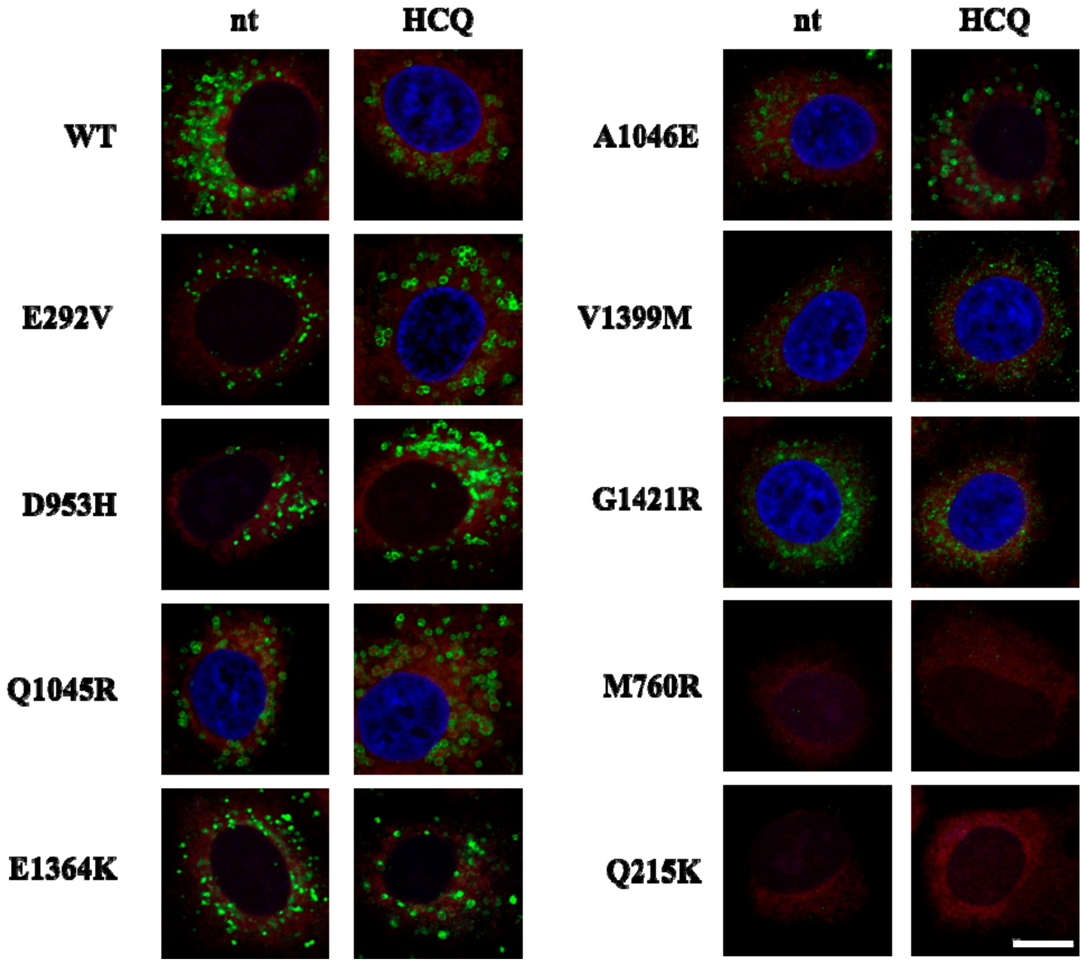
nt
HCQ
nt
HCQ
WT
E292V
D953H
Q1045R
E1364K
A1046E
V1399M
G1421R
M760R
Q215K

Figure S5

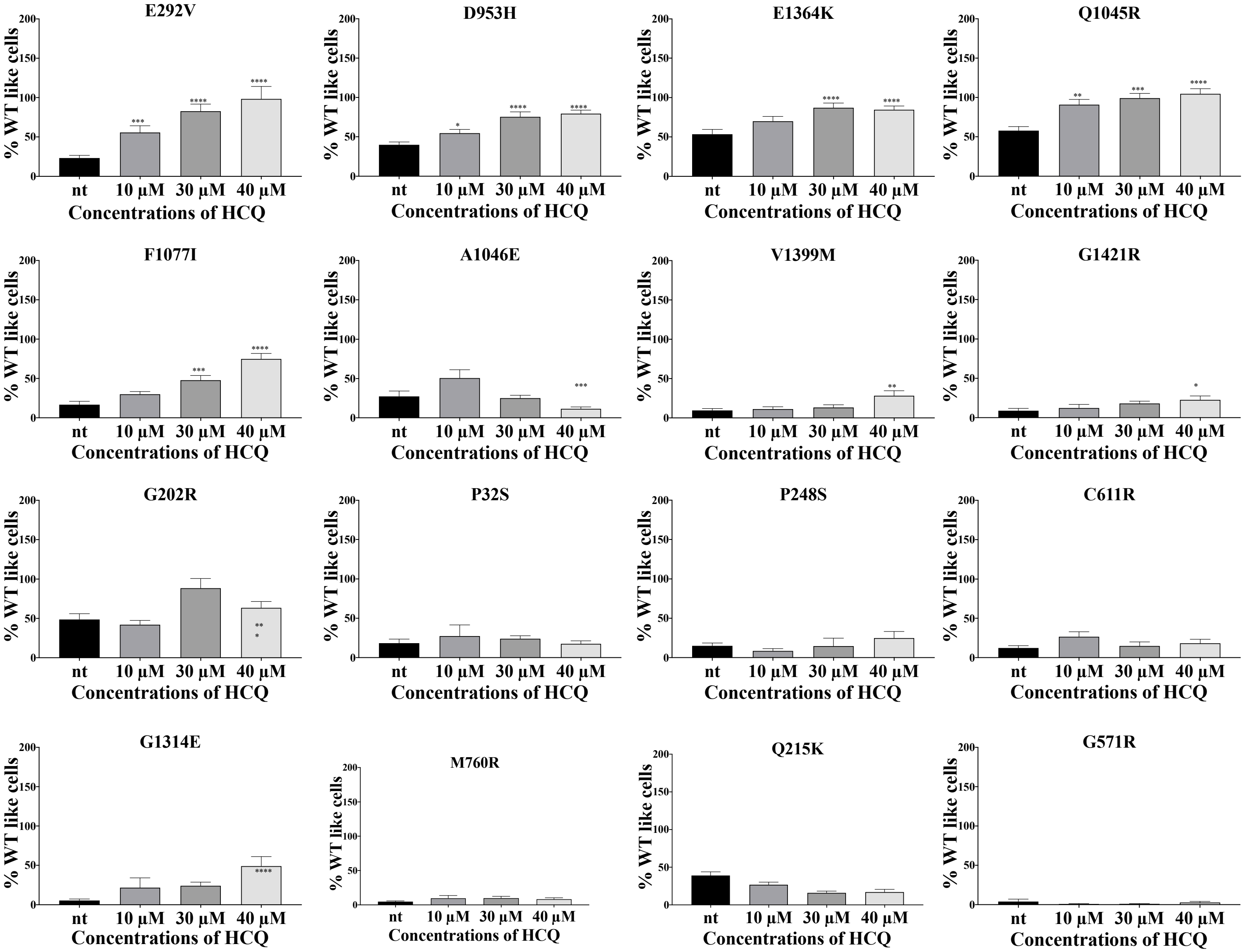
E292V
D953H
E1364K
Q1045R
F1077I
A1046E
V1399M
G1421R
G202R
P32S
P248S
C611R
G1314E
M760R
Q215K
G571R
% WT like cells
0
50
100
150
200
nt
10 µM
30 µM
40 µM
Concentrations of HCQ

Figure S6

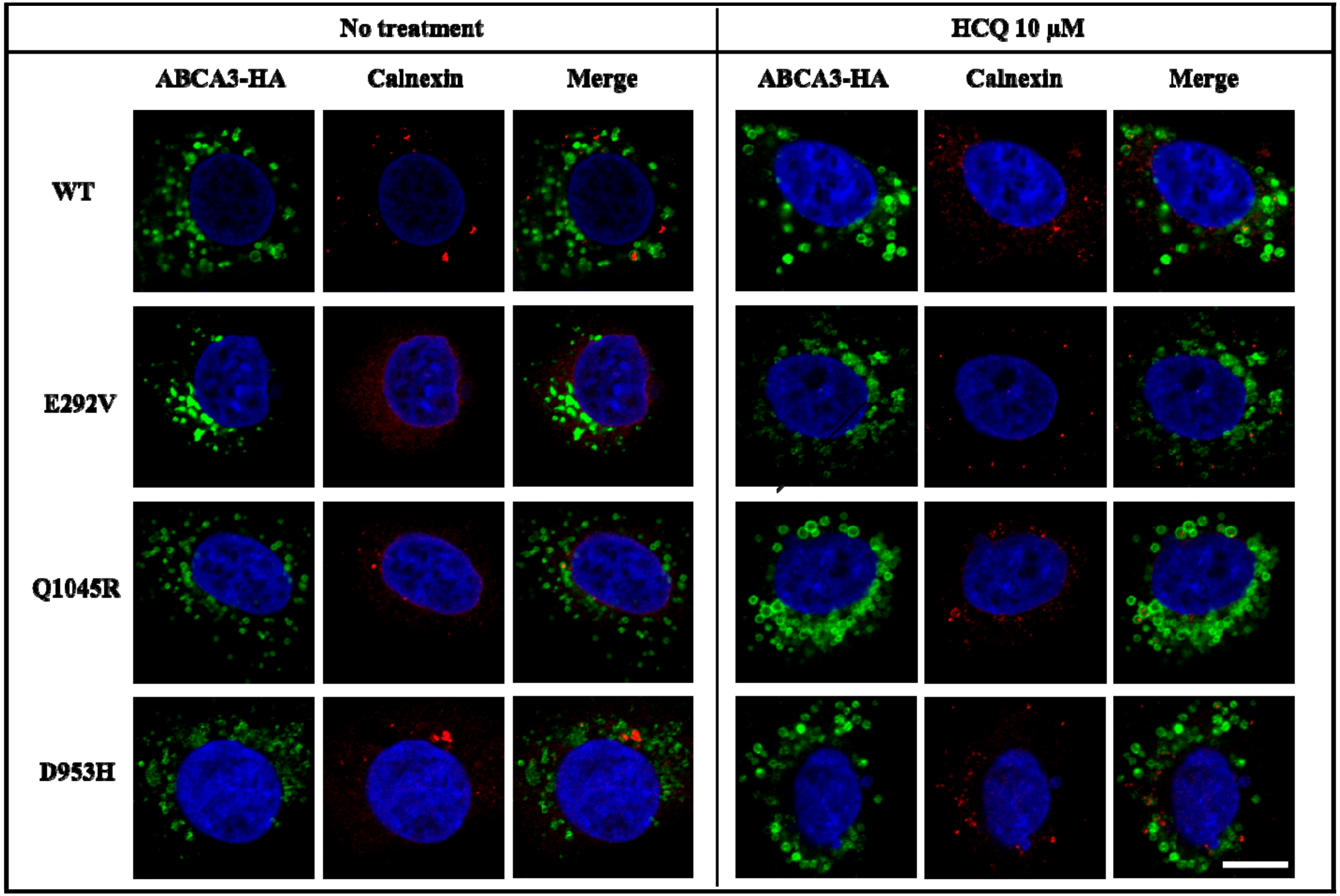

No treatment
HCQ 10 μM
ABCA3-HA
Calnexin
Merge
ABCA3-HA
Calnexin
Merge
WT
E292V
Q1045R
D953H

Reference

1. Thavagnanam, S., et al., *Variable clinical outcome of ABCA3 deficiency in two siblings.* Pediatr Pulmonol, 2013. **48**(10): p. 1035-8.
2. Yokota, T., et al., *Heterozygous ABCA3 mutation associated with non-fatal evolution of respiratory distress.* Eur J Pediatr, 2008. **167**(6): p. 691-3.
3. Hallik, M., T. Annilo, and M.L. Ilmoja, *Different course of lung disease in two siblings with novel ABCA3 mutations.* Eur J Pediatr, 2014. **173**(12): p. 1553-6.
4. Kitazawa, H., et al., *Interstitial lung disease in two brothers with novel compound heterozygous ABCA3 mutations.* Eur J Pediatr, 2013. **172**(7): p. 953-7.
5. Piersigilli, F., et al., *New ATP-binding cassette A3 mutation causing surfactant metabolism dysfunction pulmonary type 3.* Pediatr Int, 2015. **57**(5): p. 970-4.
6. Gonçalves, J.P., et al., *Novel ABCA3 mutations as a cause of respiratory distress in a term newborn.* Gene, 2014. **534**(2): p. 417-20.
7. Mitsiakos, G., et al., *A New ABCA3 Gene Mutation c.3445G>A (p.Asp1149Asn) as a Causative Agent of Newborn Lethal Respiratory Distress Syndrome.* Medicina (Kaunas), 2019. **55**(7).
8. Williamson, M. and C. Wallis, *Ten-year follow up of hydroxychloroquine treatment for ABCA3 deficiency.* Pediatr Pulmonol, 2014. **49**(3): p. 299-301.
